# Comparative analysis of the economic burdens of physical inactivity in Hungary between 2005 and 2017

**DOI:** 10.1186/s12889-020-08478-y

**Published:** 2020-08-17

**Authors:** Pongrác Ács, Antal Kovács, Dávid Paár, Márk Hoffbauer, Péter Szabó, Tünde Szabó, Miklós Stocker

**Affiliations:** 1 University of Pecs, Faculty of Health Sciences, Pecs, Hungary; 2 University of Physical Education, Budapest, Hungary; 3 Corvinus University of Budapest, Corvinus Business School, Budapest, Hungary

**Keywords:** Physical inactivity, Economic burden, PAR-method, Direct costs, Indirect burden, Population attributable risk

## Abstract

**Background:**

National economies are increasingly facing the challenge of having to finance the prevention and treatment of human diseases, and of having to compensate for the resulting loss of economic production. Physical inactivity is demonstrably closely related to the risk of developing certain disease group. Physical inactivity results in direct and indirect burdens that the present study intends to quantify in Hungary for the period between 2005 and 2017.

**Methods:**

Based on the data of the Hungarian public finances, this study determines the direct and indirect costs incurred by Hungary due to illnesses, and, through the PAR method, it quantifies the financial burden of physical inactivity incurred by the Hungarian Treasury.

**Results:**

The total financial burden of illnesses in Hungary showed a decreasing tendency from 2005 to 2017, even though the year 2017 saw an increase in costs compared to 2014. Similarly, while total public expenditure on illnesses associated with physical inactivity increased by 2017 when compared to 2009, the total amount attributable to medical conditions stemming from physical inactivity still showed a decrease of 2 billion HUF in the overall period. The biggest economic burden is posed by cardiovascular diseases, hypertension and type 2 diabetes.

**Conclusions:**

The increase in the economic burden associated with physical inactivity can be attributed to the combined effect of two factors: changes in total expenditure on specific disease groups (which showed an increase in the period under review) and changes in the physical activity levels of the Hungarian population (which showed an improvement over the period under review). Initiatives in Hungary aimed at encouraging an active lifestyle from childhood onwards should be continued since – beyond the initial impact that has already been felt to some extent in recent years - these initiatives will come to their full fruition in the coming decades.

## Background

The fundamental change towards a more sedentary lifestyle has a serious impact on people’s health. Physical inactivity is one of the most important global issues of the twenty-first century, leading to an increased risk of chronic diseases such as type 2 diabetes, cardiovascular disease, certain types of cancer (rectal, colon, breast), obesity and osteoporosis [1]. These diseases may even become the cause of death. The World Health Organization (WHO) has also identified these medical conditions as the most burdensome non-communicable diseases of today’s developed world. Regular, moderate physical activity reduces the risk of the most common of these diseases and contributes to an increased sense of well-being [2, 3]. According to the WHO, physical inactivity ranks as the fourth most significant mortality factor in the world, with 3.2 million deaths a year worldwide [4, 5].

Another study suggests that there is a lower likelihood of health problems among people engaging in regular physical activity than among those leading a sedentary lifestyle. Furthermore, there is convincing evidence that regular physical activity increases life expectancy and reduces the likelihood of developing coronary and cardiovascular problems, of suffering a stroke or developing colon cancer [6].

Inactive and sedentary lifestyles directly affect metabolism, bone mineral composition, and magnify the health effects of cardiovascular disease [7]. Furthermore, there is epidemiological evidence to suggest that a sedentary lifestyle increases the risk of cancer, obesity, metabolic and psychosocial problems [8].

According to OECD data, the average life expectancy of Hungarians at birth in 2016 was 76 years, which is 4 years below the OECD average, actually, one of the lowest on the list. For men, this value is 72.6 years, for women 79.7 years, both showing an increasing trend [9].

In recent years, the Hungarian government has made a number of efforts to bring about significant changes in the inactive lifestyle of the Hungarian population. These include measures to increase the number of physical education lessons and to improve the conditions in PE lessons at school; also the development and construction of sports facilities, increased funding for sports associations, and even the use of corporate tax incentives for sporting purposes [10]. While improving the conditions alone does not result in a change in the attitudes of the population towards sport, it is certainly a prerequisite [11].

Procedures that quantify the burden on the Hungarian economy resulting from physical inactivity are one of the ways of measuring the effectiveness of state intervention [10, 12]. This study aims to contribute to this body of research and proposes to analyse a longer time spectrum.

## Methods

To analyze the economic burden of physical inactivity, we need to start with the burden of diseases on the national economy, as physical inactivity plays a vital role in the onset of several diseases and leads to various causes of death. At a national level, diseases have direct and indirect costs.

Direct costs of diseases include treatments, medications, sick-pay allowances and associated ancilliary costs that are directly related to the illness. The direct costs in Hungary are mainly financed by the National Health Insurance Fund (NHIF) - since 2017 it is called the National Health Insurance Fund Administration (NHIFA) - but we must not disregard the cost of sick leave and private costs outside of NHIF/NHIFA financing, the latter of which are directly borne by members of society. Among the indirect burdens we include items that constitute a loss to the economy or to society as a result of the loss of work caused by a disease. There was a significant change in this area during the research period. While in 2005 and 2009 there was a long-term loss of production only in jobs on the skills-shortage list or in very special cases, by 2014 the number of job vacancies in Hungary reached 34 thousand, while by July 2017 this number rose to 73 thousand people, which is 2.4% of the workforce [13]. Our calculations were based on the following assumptions: in 2005 and 2009, in a labor market characterised by an oversupply of labor, a frictional unemployment of 6 months, group-based performance expectations, and the market of goods and services being over-supplied, people on average worked 225 days a year and loss was calculated based on GDP per capita. The study that inspired our calculations [14] had a similar calculation, but we replaced its assumptions with the above-mentioned assumptions and we broadened and tightened formulas, and corrected data that had become fact since then. However, when calculating the 2017 results, we had to change the assumptions about the labor market from the previously-outlined conditions as by 2017 Hungarian labor market had become characterized with overdemand therefore we had to increase friction time as well (8 months).

Another economic burden is presenteesim, which is the term used for the phenomenon when a sick individual goes to work, which results in poorer performance and thus loss of production.

Our main goal in this research was to quantify the economic burden of diseases for the years 2005, 2009, 2014 and 2017, and, more specifically, the costs to the national economy directly attributable to physical inactivity in the market years 2009, 2014 and 2017.

During our research, we treated as relevant secondary data Eurobarometer 2010, 2014 and 2018 and NHIF/NHIFA data from 2005, 2009, 2014 and 2017 [15–17].

In the course of our resreach we examined the types of medical conditions related to physical inactivity and their possible complications, the factual data for which was obtained from NHIF and NHIFA.

With the help of PAR method (Population Attributable Risk) - the most commonly used method in international research - we were able to obtain quantitative measurements that were used uniformly in the analysis of data for all three years:

$$ PAR=\frac{P_{\mathrm{exp}}\times \left( RR-1\right)}{1+{P}_{\mathrm{exp}}\times \left( RR-1\right)}\times 100 $$

where:

P_exp_: prevalence refers to the section of the population where a given risk is present.

RR: relative risk describes the risk associated with a sedentary lifestyle.

When using the index, it is necessary to break down the population into physically active and inactive sections, and then, by determining the relative risk rate, we can estimate the number and cost of illnesses stemming from a physically inactive lifestyle [18].

**Fig. 1:**
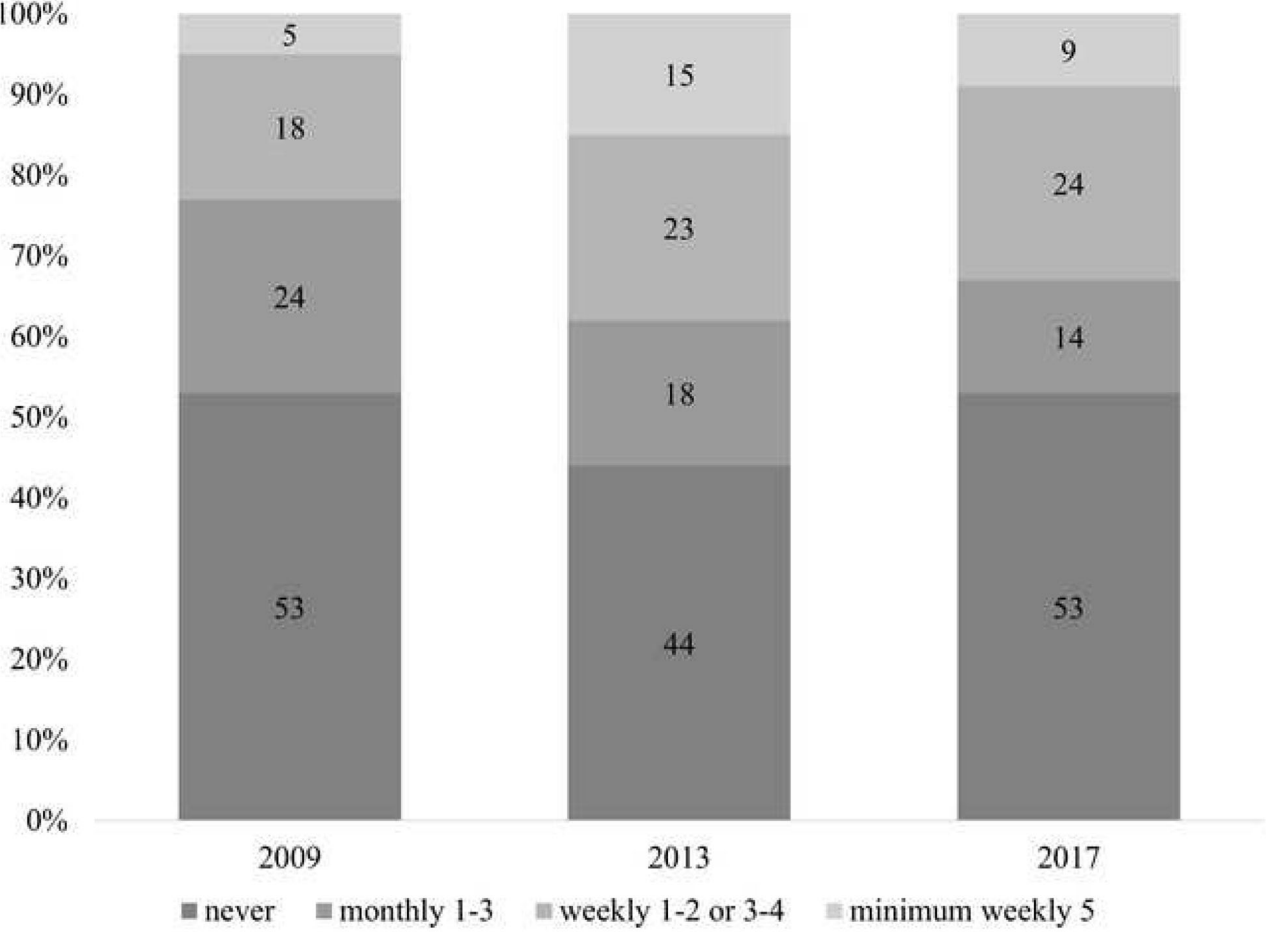
The ratio of physical activity and inactivity in Hungary in 2009–2017. Source:Special Eurobarometer 334, Special Eurobarometer 412, Special Eurobarometer 472

The physical activity indicators of the Hungarian population showed fluctuations during the period under review. The situation was the worst in 2009, when we saw 77% of the population as physically inactive (in our opinion, the health protection effect does not manifest itself in the case of those who never excercise or only do so 1–3 times a month). By 2013, this figure dropped to 62%, and at the same time, the ratio of those engaging in exercise at least 5 times per week increased threefold. In the following 4 years, a more negative trend was observed as the rate of inactive people rose to 67%, although this is still significantly better than the base figure for 2009. (Fig. 1).

With the help of meta-analysis we calculated the Relative Risk ratio (RR), an indicator which is prevalent in international literature, in order to estimate the future expenditure stemming from physical inactivity for all affected disease groups, such as cardiovascular disease, stroke, hypertension, colon cancer, type 2 diabetes, osteoporosis, depression, gastrointestinal complications, obesity, high triglyceride diseases, and deliberate self-harm [19–23].

The RR is the proportion of the applicaple diseases among people with inactive lifestyles divided by the proportion of the applicable diseases among people with active lifestyles. On the basis of the RR values, it is possible to quantify the PAR indicator by disease group for each year. (Table 1).

**Table 1:** The cumulative relative risk rate and PAR values for the examined disease types in 2009–2017

Disease types	RR	PAR 2009	PAR 2014	PAR 2017
Heart and coronary diseases	1.9	40.9	35.8	37.6
Stroke	1.4	23.5	19.9	21.1
Hypertension	1.4	23.5	19.9	21.1
Colon cancer	1.4	23.5	19.9	21.1
Type 2 diabetes	1.4	23.5	19.9	21.1
Osteoporosis	1.6	31.6	27.1	28.7
Depression	1.2	13.3	11.0	11.8
Gastrointestinal complications	1.4	23.5	19.9	21.1
Obesity	1.1	7.1	5.8	6.3
High triglycerides	1.4	23.5	19.9	21.1
Deliberate self-harm	1.1	7.1	5.8	6.3

*Source: Katzmarzyk* et al.*, 2000; Aldoori* et al.*, 1998; Ewing* et al.*, 2003; Andersen* et al.*, 2000; Schuch* et al.*, 2018*

In order to allow the data to be compared over time, the data on the burden of illnesses stemming from physical inactivity for 2014 and 2017 was recalculated to 2009 prices while the total amount of burden imposed by illnesses was recalculated to 2017 prices, using the domestic producer and consumer price index of the Hungarian Central Statistical Office (HCSO) [24].

## Results

At 2017 prices, the economic burden of illnesses amounted to more than 3753 billion forints (HUF) in 2005, of which the direct burden was 3173 billion forints. Direct costs accounted for 85% of the burden of illnesses and the 144 billion HUF sickness benefit represented just over 4.6% of total direct costs. Indirect burden represented a significantly lower percentage amounting to over 741 billion HUF. The economic burden imposed by sickness in 2005 was 11.2% of Hungary’s GDP.

By 2009, the economic burden of diseases fell to 3611 billion HUF at 2017 prices. Direct costs accounted for 85. 8% of the total burden of illnesses that year, less than 4% of which, amounting to 122 billion HUF, was for sickness allowance expenditues. Indirect burdens decreased to 644 billion HUF. The burden of sickness amounted to 11.4% of the GDP in 2009.

By 2014, the economic burden of diseases fell to 2906 billion HUF at 2017 prices. Direct costs accounted for 86% of the total burden that year and 2.8% of it were sick allowances, amounting to 70 billion forints. Indirect burdens fell to 519 billion HUF. The burden of sickness decreased to 8.7% of the GDP in 2014.

By 2017, the economic burden of illnesses increased compared to 2014, but it was still below the initial 2005 figure (HUF 3220 billion) and it decreased in comparison with the GDP. The share of direct costs dropped significantly to 78%, within which the sickness benefit represented 3.8% - to the value of 95 billion forints. At the same time, indirect burdens increased significantly to 910 billion HUF. All in all, the burden of sickness decreased to 8.4% of the GDP in 2017.

Between 2005 and 2017, the economic burden of diseases fell by 533 billion HUF, which is a total decrease of 14.2%, corresponding to an average annual decrease of 1.2% and, in the meantime, the country’s GDP increased significantly (altogether 70% at current prices). Obviously, the decrease is due to a number of reasons, but the effect of the increase in physical activity is an important factor among them. (Table 2).

**Table 2:** Economic burdens of diseases in Hungary 2005–2017 (in HUF million, in real terms in 2017)

	Economic burdens of diseases in Hungary 2005–2017	2005	2009	2014	2017	Charged to
**Direct Costs state-financed**	Medication	519,758	412,128	310,487	339,191	NHIF/NHIFA
	Medical aids	65,749	55,666	57,462	64,357	NHIF/NHIFA
	General practitioner services	93,736	93,206	99,670	122,881	NHIF/NHIFA
	Dental Services	32,313	27,204	26,952	35,531	NHIF/NHIFA
	Outpatient care	161,760	138,849	108,685	136,979	NHIF/NHIFA
	CT, MRI	16,566	16,409	17,739	25,228	NHIF/NHIFA
	Medical Centers (exluding VD clinics)	13,836	5037	0	0	NHIF/NHIFA
	Dialysis	24,992	27,541	23,798	23,171	NHIF/NHIFA
	Home Care	4598	4585	4560	5597	NHIF/NHIFA
	In-patient care	613,057	492,899	527,410	537,325	NHIF/NHIFA
	High-cost medical procedures	0	0	0	87,958	NHIF/NHIFA
	Patient transport	9351	7039	6365	7197	NHIF/NHIFA
	Spa treatments	7089	4849	4374	4085	NHIF/NHIFA
	Governmental Health Care expenditure	163,031	135,379	0	0	NHIF
	Sick Leave	144,550	121,979	70,036	95,341	NHIF/NHIFA
	Disability, rehabilitation treatment	383,409	759,107	344,626	320,603	NHIF/NHIFA
	In Total:	2,253,796	2,301,876	1,602,163	1,805,444	
**Private Costs**	Private Health Care Expenditure	755,406	658,588	797,483	545,744	Individual
	Expenditure associated with sick leave	163,697	136,950	110,551	147,039	Employer
**Indirect Costs**	Health insurance management and other costs	41,894	58,797	41,488	21,517	NHIFA
	Friction due to sickness leading to loss of production	416,157	350,154	271,346	498,013	Employer, individual, state
	*Of which: Reduced pay due to sick pay and sick leave*	82,739	70,303	74,866	102,972	Individual
	*Of which: Tax Loss for the State*	78,853	60,111	48,675	84,720	state
	Friction due to disability leading to loss of production	77,603	66,918	53,811	149,384	Society
	Presenteeism costs	44,529	37,466	29,034	53,287	Employer
	**In Total**	**3753,082**	**3,610,749**	**2,905,877**	**3220,429**	

In the 3 years examined, in the case of disease groups linked to physical inactivity the burden of illnesses on the state budget - excluding sickness allowance - amounted to 230,6 billion HUF and 286,8 billion HUF, respectively, of which the lowest value was in 2014. (However, only a part of these can be directly linked to physical inactivity, as many other risk factors play a role in the development of these diseases.) As regards the relative weight of each disease group, cardiovascular disease is the biggest burden, followed by hypertension. At the same time, type 2 diabetes was only ranked the fifth for costs in the first year, but by 2017 it became the third largest item, only slightly behind high blood pressure. Expenditure on stroke, obesity and deliberate self-harm were almost negligible compared to other disease groups. (Table 3).

**Table 3:** Total cost (incured by NHIFA (NHIF)) of those disease groups that are associated with physical inactivity and costs directly attributtable to physical inactivity itself in terms of 2009 prices (million HUF)

Disease Types	2009		2014		2017	
	Total Amount	Inactivity	Total Amount	Inactivity	Total Amount	Inactivity
Cardiovascular diseases	79,749.3	32,617.5	73,886.0	26,462.4	86,098.8	32,387.7
Stroke	436.3	102.5	294.4	58.5	270.4	57.1
Hypertension	73,083.8	17,174.7	53,629.5	10,657.2	57,741.1	12,204.0
Colon cancer	18,958.6	4455.3	11,593.7	2303.9	25,580.0	5406.5
Type 2 diabetes	21,219.6	4986.6	27,820.7	5528.5	55,326.9	11,693.7
Osteoporosis	2162.3	683.3	3933.7	1066.6	3747.8	1074.6
Depression	27,329.7	3647.1	26,693.0	2944.8	27,166.6	3210.2
Digestive disorders	13,510.8	3181.5	12,083.2	2401.1	8043.3	1700.0
Obesity	415.0	29.7	337.3	19.7	166.0	10.4
High triglycerides	32,959.2	7761.0	20,305.6	4035.1	22,609.4	4778.6
Deliberate self-harm	3.4	0.2	2.4	0.1	0.9	0.1
**Total**	**269,828.0**	**74,639.4**	**230,579.5**	**55,477.8**	**286,751.3**	**72,523.0**

Based on the results, it can be stated that in 2014 the expenditures in the state budget for the 11 disease groups examined drastically decreased by approximately 39 billion HUF, compared to the initial starting position of 269.8 billion HUF, but by 2017 the expenditures had surpassed the base total from 2009 by more than 16.9 billion HUF. Compared to 2009, only type two diabetes and osteoporosis showed an increase (31 and 82%, respectively, compared to 2009, although the latter is due to the relatively low total expenditure). For all other disease groups, the level of expenditure declined in absolute terms, resulting in a significant decrease of 39.2 billion HUF in total expenditure.

However, in the case of 2017, the picture is more varied if we examine the relative position of certain disease groups compared to 2009. Type 2 diabetes showed the most significant increase to the tune of more than 34 billion HUF. The other diseases lag behind in terms of expenditure; cardiovascular diseases and colon cancer are next with an increase of 6–6 billion forints. In addition, there is an increase in the costs associated with osteoporosis. Stagnation or decrease was observed for the other disease groups, but this could not compensate for the increase in the costs of the aforementioned diseases. The most significant drop in expenditure is observed in hypertension (15.3 billion HUF) and high triglyceride diseases (10.3 billion HUF). (Table 4).

**Table 4:** Changes in total expenditure (as reported by NHIFA (NHIF)) and changes in expenditure directly stemming from physical inactivity compared to the base level expenditure in 2009 (in real terms, adjusted to 2009 prices) (million HUF)

Disease types	2014				2017			
	Total Amount		Inactivity		Total Amount		Inactivity	
Cardiovascular diseases	− 5863.3	−7%	− 6155.1	−18.9%	6349.4	8.0%	− 229.7	−0.7%
Stroke	− 141.9	− 33%	− 44.0	− 42.9%	− 165.9	−38.0%	− 45.4	− 44.3%
Hypertension	−19,454.3	−27%	− 6517.5	−37.9%	−15,342.7	− 21.0%	− 4970.7	− 28.9%
Colon cancer	− 7364.9	−39%	− 2151.4	− 48.3%	6621.4	34.9%	951.2	21.4%
Type 2 diabetes	6601.1	31%	541.9	10.9%	34,107.3	160.7%	6707.1	134.5%
Osteoporosis	1771.4	82%	383.3	56.1%	1585.5	73.3%	391.3	57.3%
Depression	− 636.7	−2%	− 702.3	−19.3%	−163.1	−0.6%	− 437.0	− 12.0%
Digestive disorders	− 1427.7	− 11%	− 780.3	−24.5%	− 5467.5	−40.5%	− 1481.4	−46.6%
Obesity	−77.7	−19%	−10.0	−33.6%	− 248.9	−60.0%	−19.2	−64.9%
High triglycerides	−12,653.6	−38%	− 3726.0	−48.0%	− 10,349.8	−31.4%	− 2982.4	− 38.4%
Deliberate self-harm	−1.0	− 29%	− 0.1	−42.4%	− 2.5	−74.1%	− 0.2	− 77.2%
**Total**	**−39,248.5**	**−15%**	**− 19,161.6**	**− 25.7%**	**16,923.3**	**6.3%**	**− 2116.4**	**−2.8%**

Focusing on the direct burden of physical inactivity, we can conclude that 24–28% of the total expenditure of the 11 disease groups is directly attributable to physical inactivity. The major part is the cost of cardiovascular diseases and hypertension, and these were closely followed by Type 2 diabetes by 2017. Due to the fact that the total expenditure for stroke, obesity and deliberate self-harm was also insignificant compared to other disease groups, their expenditure related to physical inactivity is insignificant. In the case of deliberate self-harm, the costs cannot be measured even in the order of one hundred thousand forints. (Table 3).

Compared to 2009, the decrease for the year 2014 of the direct costs stemming from physical inactivity is larger in proportion than the decrease of total expenditure. This is true of each disease group, and for those two groups (type 2 diabetes and osteoporosis) where there was an actual increase in costs, the increase was less for physical inactivity-related expenses than for overall expenses. The largest drop in monetary terms can be observed in the case of hypertension and cardiovascular diseases (over 6 billion HUF), but there was a decrease of 3.7 and 2.2 billion HUF, respectively, in high triglyceride-related diseases and colon cancer. However, percentage-wise, NHIF achieved the highest cost reduction for high triglycerides and colon cancer (48%), closely followed by a decrease in stroke expenditure (42.9%) and deliberate self-harm (42.4%). - although in the last two categories, the low sum total spent also makes this decrease appear larger percentage-wise than would be the case with larger totals.

Compared to 2009, the expenditure related to physical inactivity in 2017 shows a decrease of 2.1 billion HUF. At the level of the individual disease groups, the amounts vary, the most significant decline in absolute terms is in the high blood pressure and high triglyceride-related illness groups. However, the burden of type 2 diabetes increased significantly and there was an increase in colon cancer and osteoporosis disease groups. The direction and extent of the changes are mostly comparable to the total expenditure amounts at the overall level of the disease groups, although the changes in the physical inactivity rate naturally lead to differences in the specific values. This is so much so that the total expenditure amounts increased at the level of all disease groups (by 6.3%), but overall the expenditure related to physical inactivity shows a decrease of 2.8%. (Table 4).

## Discussion

We can clearly conclude, similarly to other international researches [19, 25–27] that physical activity and forms of recreational exercise have a protective effect (e.g.: a preventive effect against certain types of chronic diseases, cardiovascular, locomotor disorders, diabetes and certain types of tumors). The decrease in physical inactivity has a positive effect on productivity, as fewer people avail themselves of sick leave. A study of economic development over the past century has concluded that the advancement of the population’s health status is responsible for about 30–40% of economic growth [28–30].

In our comparative study, we used four sampling points between 2005 and 2017, to demonstrate the burden of diseases at the level of the national economy for the various load-carriers. In the period under review, the economic burden decreased significantly overall; from 11.2% of the GDP to 8.4%. The weight of indirect burden increased, however, as in the currently demand-dominated labor-market it is more difficult to replace lost workforce. In the period of analysis the number of employees in Hungary increased with 20% which increased the amount of sick leave and number of sickness days but their GDP contribution was significantly higher. Although associated costs and burdens increased in nominal terms, they decreased in relation to the GDP.

A large part of diseases’ burdens are borne by the state and society (64%), followed by households (20%) and employers (16%). The proportions are similar to Ding et al. in European countries (included Hungary) [31], although we estimate that the burdens on employers are higher and the burdens on households are lower in Hungary.

Since 2009, the physical activity rate of the Hungarian population has been fluctuating, but overall there is an improving tendency, which is also apparent in the savings potential of the examined expenditures categories compared to the GDP. The amount of spending depends heavily, apart from the physical inactivity rate, on the number of employees as well, as those people who are not employed can not have sick leave, for example. Overall government spending depends on the budget of the country which is connected to the overall economic situation.

Cardiovascular diseases accounts for most of the cost of physical inactivity in Hungary, which coincides with Mattli et al. in Switzerland [32]. However 26% of direct inactivity costs are attributable to depression in Switzerland, while NHIFA’s depression costs account for only 4–5% in Hungary.

In Hungary, a number of measures have recently been taken in order to integrate physical activity and sport into people’s daily lives. Such measures include the introduction of everyday physical education in schools or the extensive development of sports infrastructure [33]. However, the effects of these measures will have a more pronounced effect in the long run. Several studies have shown that high physical activity in childhood is not yet measurable in terms of economic returns (less frequent use of health care and a lower cost associated with using them), as some effects – such as the high cost of sports injuries, high rates of childhood illness – have a negative bearing on the rate of return on investment. However, a long-term change of attitude and openness to physical activity at later stages in life are where these measures bear a profit, so any effort to support childhood sports is rewarding [34, 35]. In addition, international research data confirm the fact that those parents who are themselves engaged in sport or currently do so are more likely to prefer sporting activities among their children. It is important to draw attention to the fact even minimal physical activity has a health-improving effect at any stage of life [36]. That is why sport and health policies at all times should promote recreational activities for all ages, not just young people.

We would like to expand the scope of our current research if we could also examine how the patient numbers varied each year by disease group, unfortunately, the data was not available. This would be of particular interest for the year 2017, as the expenditure on illnesses showed a significant increase in real terms compared to 2009, which may be due to the fact that more patients received care and treatment, but may also be due an increase of the normative provision per person by the government, possibly to provide better quality care.

If we posit, based on the Eurobarometer data, that the physical activity rate improved compared to 2009, we could also assume that fewer people were treated for the analysed medical conditions in 2017, which would basically have a downward effect on total expenditure. At the same time, however, the picture is somewhat shaded by the fact that even if the attitude of the population towards regular physical activity has changed in the last few years, it is not certain that the number of illnesses would decrease significantly in such a short period, as the negative effects of a sedentary lifestyle led for decades would not be easily offset by a few years of excercise-laden lifestyle [37]. This is especially true for older age groups. That is to say, a reduction in the number of patients is not realised yet in patient care. At the same time, the use of rapidly-developing medical technologies is also increasing the financial burden on the budget, as the higher costs of new technologies make medical care per patient more expensive. On the other hand, it should not be forgotten that healing can be made more effective and can lead to higher returns on human capital.

## Conclusion

The study examined the development and composition of direct and indirect burdens of disease in Hungary and the costs of physical inactivity to the state budget. These burdens fell in the examined periode which was associated with an increase of GDP.

However there was an increase in the economic burden associated with physical inactivity which can be attributed to the combined effect of two factors: changes in total expenditure on specific disease groups (which showed an increase in the period under review) and changes in the physical activity levels of the Hungarian population (which showed an improvement over the period under review). Initiatives in Hungary aimed at encouraging an active lifestyle from childhood onwards should be continued since – beyond the initial impact that has already been felt to some extent in recent years - these initiatives will come to their full fruition in the coming decades.

## Data Availability

The data of the state financed direct costs that support the findings of this study are available from National Health Insurance Fund Administration but restrictions apply to the availability of these data, which were used under license for the current study, and so are not publicly available. Data are however available from the authors upon reasonable request and with permission of National Health Insurance Fund. The datasets of the private ind indirect costs used and analysed during the current study are available from the corresponding author on reasonable request.

## Abbreviations

CT: Computed Tomography
GDP: Gross Domestic Product
HCSO: Hungarian Central Statistical Office
HUF: Hungarian Forint
MRI: Magnetic Resonance Imaging
NHIF: National Health Insurance Fund
NHIFA: National Health Insurance Fund Administration
OECD: Organisation for Economic Co-operation and Development
PAR: Population Attributable Risk
RR: Relative Risk
VD: Veneral Disease
WHO: World Health Organization
